# TrkB interacts with ErbB4 and regulates NRG1-induced NR2B phosphorylation in cortical neurons before synaptogenesis

**DOI:** 10.1186/s12964-014-0047-9

**Published:** 2014-07-24

**Authors:** Chirayu D Pandya, Anilkumar Pillai

**Affiliations:** 1 Department of Psychiatry and Health Behavior, Medical College of Georgia, Georgia Regents University, 997 St. Sebastian Way, Augusta, GA, USA

**Keywords:** NRG1, BDNF, TrkB, Neurons, Synapse, NR2B

## Abstract

**Background:**

Neuregulin 1 (NRG1) and NMDARs play important roles in various neuronal functions including neural development. NMDARs also promote many cellular events such as proliferation and survival of neuroblasts before synapse formation. Although many recent studies have indicated that NRG1 regulates NMDAR function in cortical neurons, the effect of NRG1 on NMDAR activation before synapse formation is not well studied.

**Results:**

NRG1 induces activation of NMDAR subunit NR2B, and tropomyosin-related kinase receptor B (TrkB), the receptor for BDNF via activation of phospholipase C-gamma (PLC-γ) in immature primary cortical neurons. Our data using TrkB inhibitor (K252a), TrkB siRNA and TrkB−/− neurons demonstrated that TrkB inhibition suppresses NRG1-induced NR2B activation in neurons. We found that NRG1 stimulation leads to GABA_A_ receptor-mediated TrkB activation. Co-immunoprecipitation and proximity ligase assay showed that TrkB interacts with ErbB4 (NRG1 receptor) and the TrkB-ErbB4 interaction was increased following NRG1 treatment. A significant reduction in TrkB-ErbB4 interaction was observed in the prefrontal cortex of schizophrenia subjects. We found significant increase in released BDNF levels following NRG1 treatment, which was inhibited by ErbB4 inhibitor, AG1478. In addition, pretreatment with BDNF neutralizing antibody, but not control IgG abolished NRG1-induced increases in phospho-TrkB and phospho-NR2B levels. Moreover, studies using TrkB mutants showed that intercellular domain of TrkB is necessary for TrkB-ErbB4 interaction and NR2B activation.

**Conclusions:**

BDNF/TrkB signaling plays an important role in the NRG1-stimulated NR2B regulation. These findings could be of relevance to many neurodevelopmental disorders, as NRG1 and BDNF signaling pathways have been implicated in autism and schizophrenia.

## Introduction

Activation of NMDA type glutamate receptors (NMDARs) facilitate a number of signaling pathways involved in neuronal development, learning, and memory [1]. The developmentally regulated expression of NR2 subunits is a key component to controlling normal development of synapses [2]. Moreover, the NMDAR composition changes through development, with NR2B dominating in immature neurons [3]. Interestingly, prior to synapse formation, activation of NMDARs promotes many cellular events including proliferation and survival of neuroblasts [4]. However, the regulatory mechanisms of NMDAR activation are less investigated before synaptogenesis than during or after synaptogenesis.

Although the roles of a number of brain-derived trophic molecules have been implicated in neuroplasticity, recent studies show that neuregulin 1 (NRG1) plays a major role in neurodevelopment and pathophysiology of neuropsychiatric disorders [5]. NRG1 is a member of the neuregulin family of four related genes (NRG1-4) and is synthesized as a transmembrane protein, which then undergoes proteolytic processing by both neuronal activity and interaction with its ErbB receptor, ErbB4 [6]. NRG1 is widely expressed throughout development and adulthood, and plays important roles in neural development including neuron migration, axon projection, myelination, and neurotransmitter receptor maintenance [5]. Recent studies have found the role of NRG1 in the regulation of glutamatergic signaling; in particular NR2B function [7]-[9]. In addition, PLCgamma/Ca^2+^ signaling is known to mediate NRG1-induced NMDAR regulation in neurons [10]. However, the above studies have investigated the effects of NRG1 on NR2B activation either in neuroblastoma cell lines or in neurons after synaptogenesis. Moreover, NRG1 has been shown to promote excitatory synapse development in GABAergic interneurons [11]. These studies indicate that the effect of NRG1 on NR2B function in neurons prior to synapse formation needs further investigation.

Brain derived neurotrophic factor (BDNF) is a neurotrophic molecule that plays very important roles in neurodevelopment and adult brain plasticity [12]. It is known that binding of BDNF to TrkB elicits various intracellular signaling pathways, including phospholipase Cγ (PLCγ), which mediate the neuroprotective effects of BDNF [13]. Moreover, BDNF enhances NR2B mediated synaptic transmission via activation of TrkB [14]. Interestingly, postmortem studies have reported alterations in BDNF, NRG1 and their receptors in prefrontal cortex of schizophrenia subjects indicating their roles in the pathophysiology of this disorder [15]-[17]. Moreover, accumulating evidence has suggested alterations in glutamatergic transmission via NMDA receptors in schizophrenia [18].

Based on the above studies that both NRG1 and BDNF regulate neural development, and NMDARs promotes many neuronal functions before synaptogenesis, we hypothesized that NRG1-induces NR2B activation in immature neurons from embryonic mouse cortex via BDNF/TrkB dependent mechanism. We report that TrkB inhibition suppressed NRG1-induced NR2B phosphorylation in neurons. We found that the interaction between ErbB4 and TrkB plays an important role in NRG1 regulation of NR2B.

## Results

### TrkB inhibition suppresses NRG1-induced NR2B phosphorylation in primary cortical neurons

**Figure 1:**
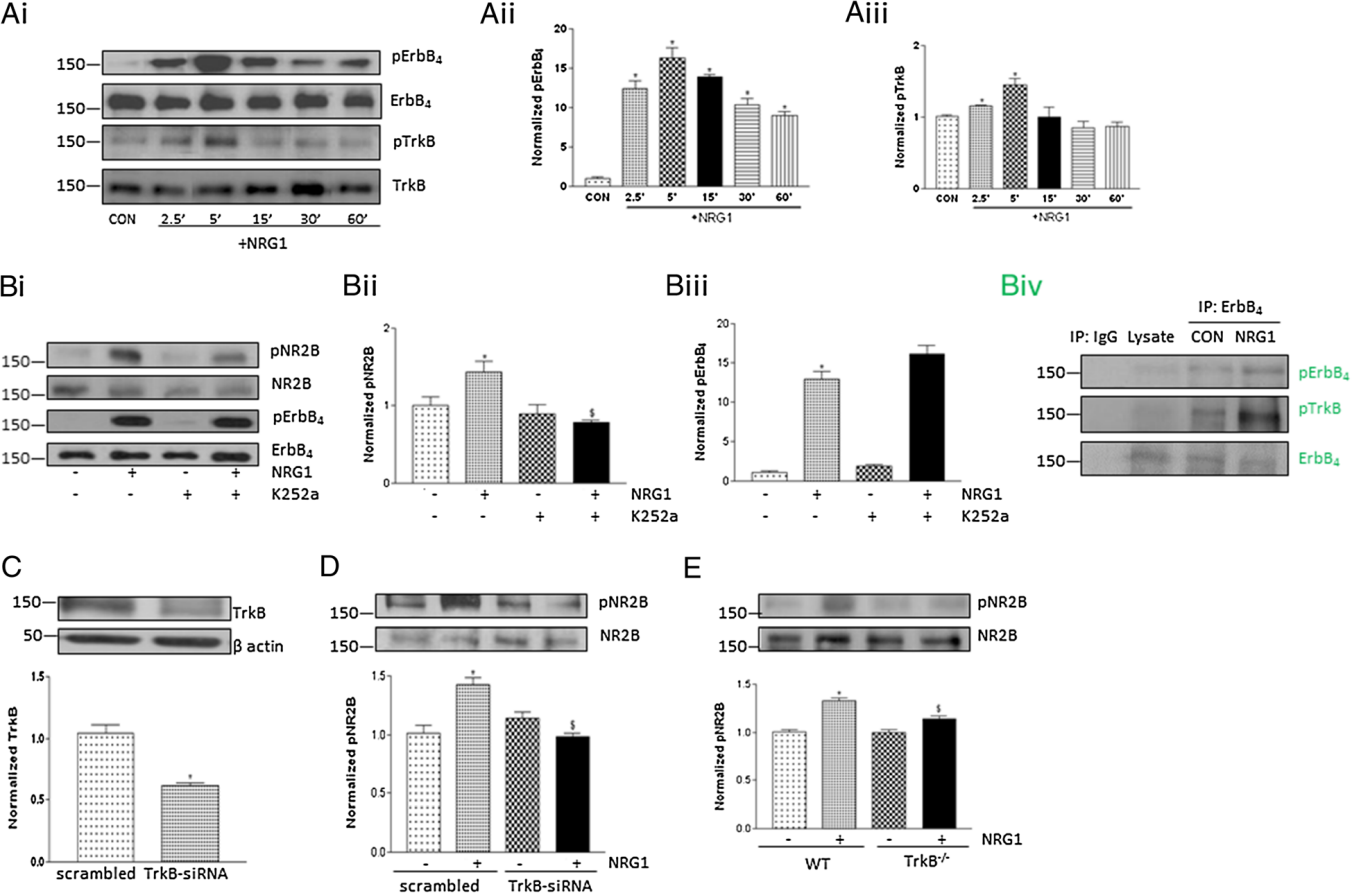
**Inhibition of TrkB suppressed NRG1 induced activation of NR2B in primary cortical neurons. (Ai)** Time dependent effect of NRG1 on ErbB_4_ and TrkB receptor activation. NRG1 (5 nM) was applied at DIV4 for indicated time course. **(Aii)** pErbB_4_ values were normalized against total ErbB_4_ levels and **(Aiii)** pTrkB values were normalized against total TrkB levels. Data represent mean ± SEM. (n = 3). *P < 0.01 vs control (CON). **(Bi)** Trk inhibitor K252a blocks the stimulatory effect of NRG1 on NR2B activation, but not ErbB_4_ activation in cortical neurons. Cortical neurons were pretreated with 100 nM K252a for 30 min, followed by NRG1 for 5 min. **(Bii)** Quantification of normalized pNR2B and **(Biii)** pErbB_4_. Data represent mean ± SEM. (n = 5). *P < 0.01 vs control; ^$^P< 0.01 vs NRG1 treatment. **(Biv)** The specificity of the pErbB4 antibody was tested in cortical neurons. Immunoprecipitation (IP) with the anti-ErbB4 antibody or control IgG antibody followed by blotting with the anti-pErbB4, anti-pTrkB or anti-ErbB4 antibody was performed in neurons (DIV4) after NRG1 stimulation. **(C)** Endogenous TrkB was decreased after TrkB-siRNA transfection compared to scrambled siRNA. siRNA was transfected 48 h before lysates were collected. Data represent mean ± SEM. (n = 3). *P < 0.05. **(D)** NRG1-induced NR2B activation was reduced in TrkB-siRNA transfected cultures. Data represent mean ± SEM. (n = 3). *P < 0.05 vs scrambled siRNA group; ^$^P < 0.05 vs NRG1 treated scrambled siRNA group. **(E)** Immunoblotting analysis of NRG1-induced NR2B activation in primary cortical neurons isolated from WT and TrkB−/− mice. Data represent mean ± SEM. (n = 3). *P < 0.05 vs WT control; ^$^P < 0.05 vs WT NRG1 treatment.

We first determined the effect of NRG1 on the activation of ErbB4, TrkB and NR2B in dissociated cortical neurons before synapses are formed (DIV 4) [19]. Acute administration of NRG1 increased ErbB4 phosphorylation at Tyr1284 in cortical neurons (Figure 1A*i* and *ii*). In addition, TrkB phosphorylation at Y816 was increased as a result of NRG1 treatment (Figure 1A*i* and *iii*). We observed increased NR2B phosphorylation at Ser1303 in neurons following NRG1 exposure (Figure 1B*i*)*.* Next, we examined NRG1-induced NR2B phosphorylation in cultured cortical neurons after exposure to K252a (a Trk inhibitor). K252a (30 min) suppressed NRG1-induced NR2B phosphorylation indicating that NR2B regulation by NRG1 involved Trk receptor activity (Figure 1B*i* and *ii*). Trk inhibition did not alter NRG1-induced ErbB4 activation (Figure 1B*i* and *iii*). The specificity of the phosphoErbB4 antibody was confirmed in neuronal lysates by immunoprecipitation of ErbB4 followed by immunoblotting with phosphoErbB4 or phosphoTrkB antibody (Figure 1B*iv*). To verify the role of TrkB, we used TrkB siRNA to silence TrkB expression in primary cortical neurons (Figure 1C). The downregulation of TrkB by TrkB siRNA abolished NRG1-induced NR2B phosphorylation (Figure 1D). As a negative control, a scrambled siRNA was used, which permitted NR2B activation following NRG1 treatment. To validate the above results further, we used primary cortical neurons from TrkB knockout (TrkB−/−) mice. Embryonic cortical neurons isolated at E16 from TrkB−/− and wild-type (WT) mice were treated at DIV 4 with NRG1. NR2B activation was abolished in TrkB−/− neurons (Figure 1E). These results suggest that NRG1-induced NR2B phosphorylation occurs via TrkB signaling.

### BDNF mediates NR2B phosphorylation by NRG1

**Figure 2:**
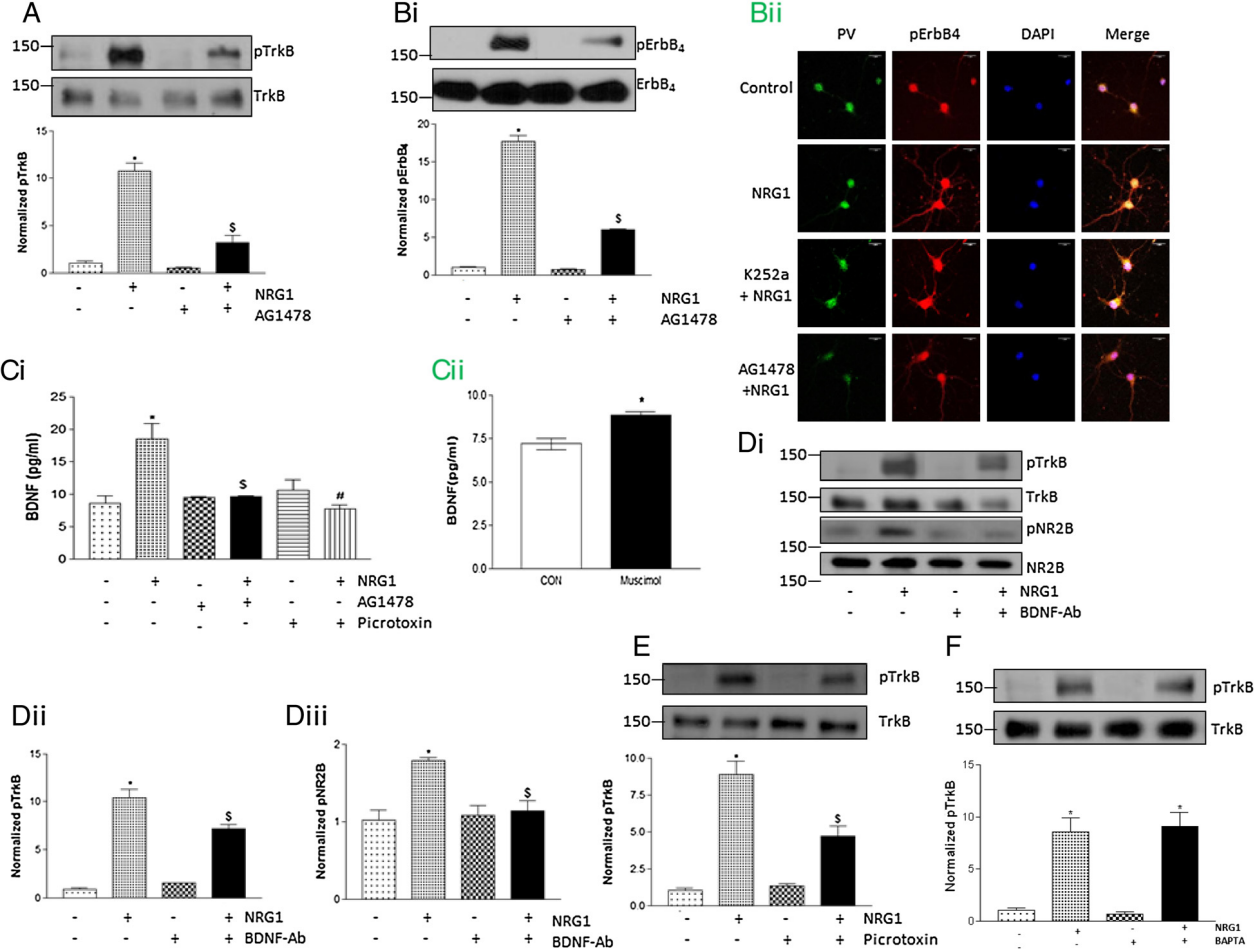
**BDNF is involved in NR2B phosphorylation by NRG1. (A)** NRG1-induced TrkB activation was reduced by pre-treatment with ErbB_4_ inhibitor, AG1478 (5 μM) for 20 min. The upper panel shows a representative autoradiogram of pTrkB and TrkB, and the lower panel represents normalized pTrkB values. **(Bi)** Immunoblot analysis shows ErbB4 activation with NRG1, which was blocked by AG1478. The upper panel shows a representative autoradiogram of pErbB4 and ErbB4, and the lower panel represents normalized pErbB4 values. **(Bii)** NRG1 induced activation of ErbB4 was inhibited by AG1478, but not by K252a. Cortical neurons were treated with 5 μM AG1478, an inhibitor of ErbB4, or K252a, an inhibitor of TrkB, for 20 min prior to the addition of NRG1. Neurons were fixed and stained with phospho-ErbB4 and parvalbumin (PV) antibodies, and visualized with Cy3 (red) and Cy2 (green) coupled secondary antibodies, respectively. Scale bar, 20 μm. **(Ci)** NRG1-induced increase in BDNF levels in culture medium was blocked by pre-treatment of AG1478 or Picrotoxin (100 μM; 30 min pre-treatment). **(Cii)** GABA agonist, muscimol (50 μM; 30 min pre-treatment) increased BDNF levels in culture medium. **(Di)** Immunoblot analysis of NRG1-induced activation of TrkB and NR2B, in presence of BDNF neutralizing antibody (1 μg/ml) or control IgG antibody pre-treated for 30 min. Quantification of **(Dii)** pTrkB and **(Diii)** pNR2B showed a significant reduction in their levels with BDNF neutralizing antibody. **(E)** Inhibition of GABA_A_ receptor activity with Picrotoxin suppresses NRG1-induced activation of TrkB. The upper panel shows a representative autoradiogram of pTrkB and TrkB, and the lower panel represents normalized pTrkB values. **(F)** Chelating intracellular Ca2+ with BAPTA-AM (50 μM) has no effect on NRG1-induced activation of TrkB. Data represent mean ± SEM. (n = 3). *P < 0.05 vs WT control; $P < 0.05 vs WT NRG1 treatment.

Since TrkB was found as a key mediator of NR2B regulation by NRG1, the possible role of its ligand, BDNF was investigated. We hypothesized that increase in BDNF release following NRG1 treatment stimulates NR2B phosphorylation. We found a significant increase in TrkB phosphorylation in cultured cortical neurons following NRG1 treatment, which was blocked by AG1478, the ErbB4 kinase inhibitor (Figure 2A). Western blot analysis of phosphoErbB4 confirmed the effectiveness of AG1478 in inhibiting ErbB4 phosphorylation (Figure 2B*i*). Immunofluorescence analysis of phosphoErbB4 and parvalbumin showed that NRG1 induced ErbB4 activation occurs in GABAergic interneurons (Figure 2B*ii*). Also, immunofluorescence data confirmed our western blot results that NRG1-induced ErbB4 activation is not dependent on TrkB activation status. Moreover, BDNF ELISA showed a significant increase in released BDNF levels following NRG1 treatment, which was inhibited by AG1478 (Figure 2C*i*). In addition, pretreatment with BDNF neutralizing antibody (1 μg/ml), but not control IgG abolished NRG1-induced increases in phospho-TrkB (Figure 2D*i* and *ii*) and phospho-NR2B (Figure 2D*i* and *iii*) levels. We observed reduction in total TrkB levels in neurons treated with BDNF neutralizing antibody as compared to control IgG-treated neurons (Figure 2D*i*). The reduction in TrkB levels might be due to the cellular response to the lack of ligand (BDNF) in neurons treated with BDNF neutralizing antibody. Overall, these results support the role of BDNF/TrkB signaling in the regulation of NR2B phosphorylation by NRG1 in neurons.

It has been shown that neuregulin can induce GABA_A_ receptor expression in neurons [20], and GABA_A_ receptor activation stimulates BDNF release in developing neurons [21]. Since we found a significant role of BDNF in mediating NRG1-induced TrkB activation, we examined whether inhibition of GABA_A_ receptor activity could block the effect of NRG1 on TrkB phosphorylation. We found a significant inhibition on NRG1-induced BDNF release (Figure 2C*i*) as well as TrkB phosphorylation (Figure 2E) in neurons pretreated with picrotoxin, a GABA_A_ receptor antagonist. However, treatment with a GABA agonist, muscimol (50 μM) significantly increased BDNF release (Figure 2C*ii*). Furthermore, although calcium has been shown to mediate an important role in BDNF release, chelating intracellular calcium by BAPTA-AM did not inhibit NRG1-induced TrkB phosphorylation in neurons, indicating that calcium is not a key mediator in NRG1-induced TrkB activation (Figure 2F).

### PLCγ is involved NR2B phosphorylation by NRG1

**Figure 3:**
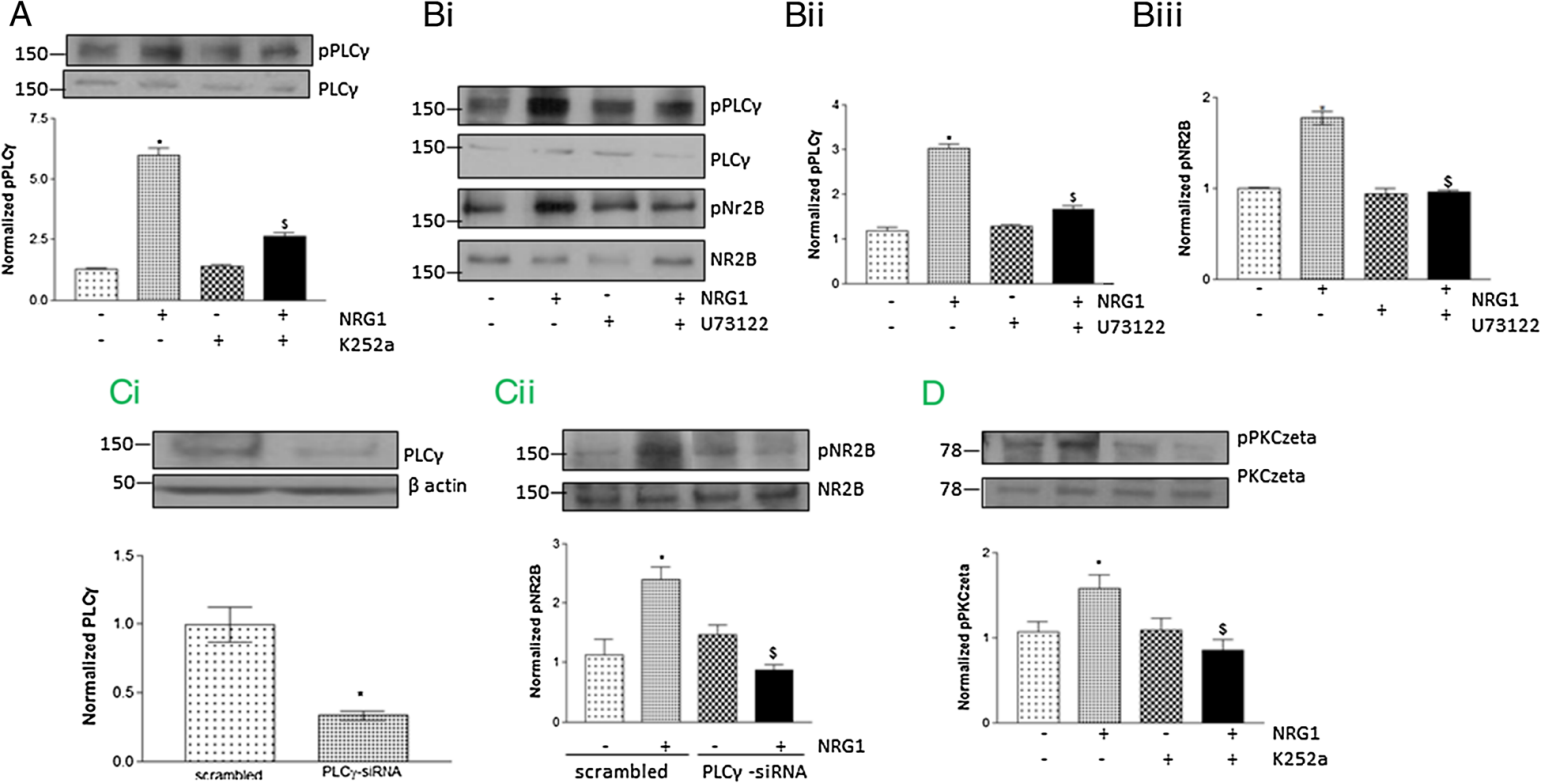
**PLCγ mediates NR2B activation by NRG1. (A)** NRG1-induced PLCγ activation is TrkB dependent. The upper panel shows a representative autoradiogram of pPLCγ and PLCγ, and the lower panel represents normalized pPLCγ values. **(Bi)** Immunoblot analysis of NRG1-induced activation of PLCγ and NR2B in presence of U73122. U73122 inhibited NRG1-increased phosphorylation of **(Bii)** PLCγ and **(Biii)** NR2B. Data represent mean ± SEM. (n = 5). *P < 0.05 vs control; ^$^P < 0.05 vs NRG1 treatment. **(Ci)** Endogenous PLCγ was decreased after PLCγ siRNA transfection compared to scrambled siRNA. The upper panel shows a representative autoradiogram of PLCγ and β-actin, and the lower panel represents normalized PLCγ values. Data represent mean ± SEM. (n = 3). *P < 0.05. **(Cii)** NRG1-induced NR2B activation was reduced in PLCγ siRNA transfected cultures. The upper panel shows a representative autoradiogram of pNR2B and NR2B, and the lower panel represents normalized pNR2B values. Data represent mean ± SEM. (n = 3). *P < 0.05 vs scrambled siRNA group; ^$^P < 0.05 vs NRG1 treated scrambled siRNA group. **(D)** Immunoblotting analysis of NRG1-induced PKCzeta activation in isolated primary cortical neurons. The upper panel shows a representative autoradiogram of pPKCzeta and PKCzeta, and the lower panel represents normalized pPKCzeta values. Data represent mean ± SEM. (n = 3). *P < 0.05 vs control; ^$^P < 0.05 vs NRG1 treatment.

PLCγ plays a key role in tyrosine kinase-mediated signaling pathways. Moreover, NR2B is known to bind to the SH domain of PLCγ facilitating NR2B phosphorylation [22]. Therefore, we hypothesized that TrkB mediated NRG1-induced NR2B phosphorylation requires PLCγ activation. We found a significant increase in PLCγ activation (as determined by phosphorylation at Tyr783) in cultured neurons following NRG1 treatment, which was abolished by pretreatment with K252a (Figure 3A). Moreover, pre-treatment with PLCγ inhibitor U73122 dramatically reduced NRG1-stimulated PLCγ (Figure 3B*i and ii*) as well as NR2B (Figure 3B*i* and *iii*) phosphorylation in neurons suggesting a potential role of PLCγ in NRG1-induced NR2B activation. The role of PLCγ was further confirmed using PLCγ siRNA in primary cortical neurons (Figure 3Ci). The downregulation of PLCγ by specific siRNA abolished NRG1-induced NR2B phosphorylation as compared to scrambled siRNA (Figure 3C*ii*). Since we did not find any significant effect of BAPTA-AM on NRG1-induced TrkB phosphorylation in neurons, we examined the role of atypical PKC species in the above experiment. We found that NRG1 treatment induced phosphorylation of PKCzeta, an atypical PKC species in primary cortical neurons, and the above effect was inhibited by pretreatment with K252a (Figure 3D).

### TrkB-ErbB4 interaction is involved in NR2B activation by NRG1

**Figure 4:**
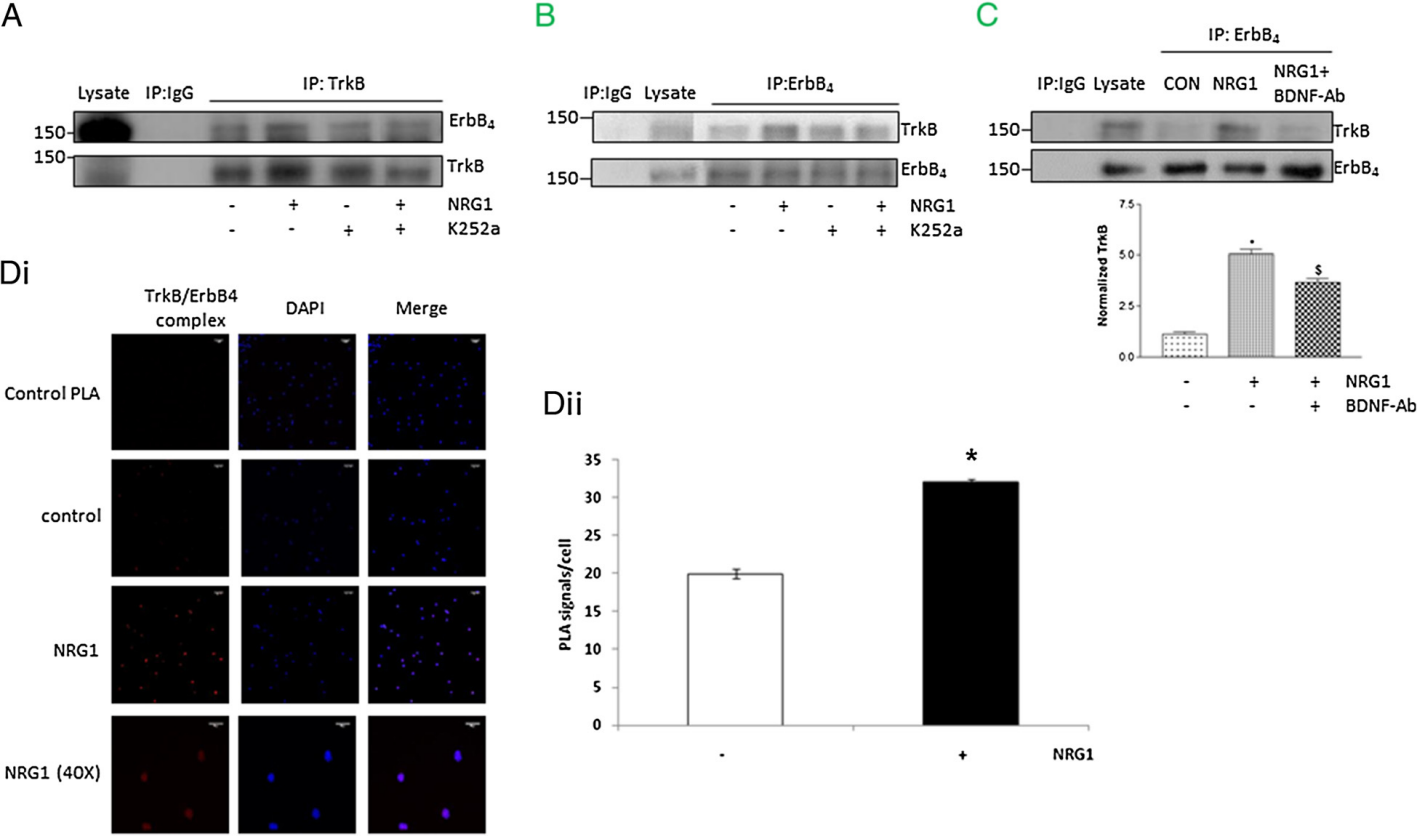
**TrkB-ErbB4 interaction was increased following NRG1 treatment. (A)** Immunoprecipitation (IP) with the anti-TrkB antibody or control IgG antibody followed by blotting with the anti-ErbB4 or anti-TrkB antibody was performed in neurons (DIV4) after NRG1 stimulation with or without K252a pretreatment. **(B)** IP with the anti-ErbB_4_ antibody or control IgG antibody followed by blotting with the anti-TrkB or anti-ErbB4 antibody. **(C)** Inhibition of NRG1-induced increase in TrkB-ErbB4 interaction in neurons (DIV4) with a BDNF neutralizing antibody. The upper panel shows a representative autoradiogram of TrkB and ErbB4, and the lower panel represents normalized TrkB values. Data represent mean ± SEM. (n = 3). *P < 0.05 vs control; ^$^P < 0.05 vs NRG1 treatment. **(Di)** PLA was performed in DIV4 cortical neurons following treatment with NRG1 for 5 min. The antibodies were omitted in the control PLA group (control PLA). The images were acquired at 20X or 40X and scale bar is 20 μM. The red spots represent the interaction between TrkB and ErbB4. **(Dii)** Quantitation of PLA signal was performed in three independent experiments using imageJ. Data represents mean PLA signal/cell ± SEM. *P < 0.05 vs control.

To understand the mechanism via which TrkB controls NRG1-induced NR2B phosphorylation, the endogenous levels of TrkB and ErbB4 after NRG1 treatment was examined. No change in the above proteins was found in the western blot analysis (Figure 1A*i*). Next, the possible interaction between ErbB4 and TrkB was investigated in neurons following NRG1 exposure. Following immunoprecipitation with anti-TrkB antibody, coprecipitated ErbB4 was found in neuronal lysates (Figure 4A). NRG1 treatment significantly increased the coprecipitated ErbB4, whereas pretreatment with K252a abolished the interaction. Reverse immunoprecipitation also showed similar results (Figure 4B). In addition, pretreatment with BDNF neutralizing antibody abolished NRG1-induced increases in TrkB-ErbB4 complex formation (Figure 4C). The interaction between TrkB and ErbB4 was further confirmed by PLA. The PLA identifies interaction between two proteins in their native form and results in a fluorescent signal in the form of a spot when the two proteins of interest are closer than 40 nm. There was a significant increase in PLA signal in NRG1-stimulated neurons compared with unstimulated cells (Figure 4D*i* and *ii*). Taken together, these results indicate that NRG1-induced increase in TrkB-ErbB4 complex depends on BDNF/TrkB signaling.

### The intracellular domain of TrkB Contributes to ErbB4-TrkB interaction

**Figure 5:**
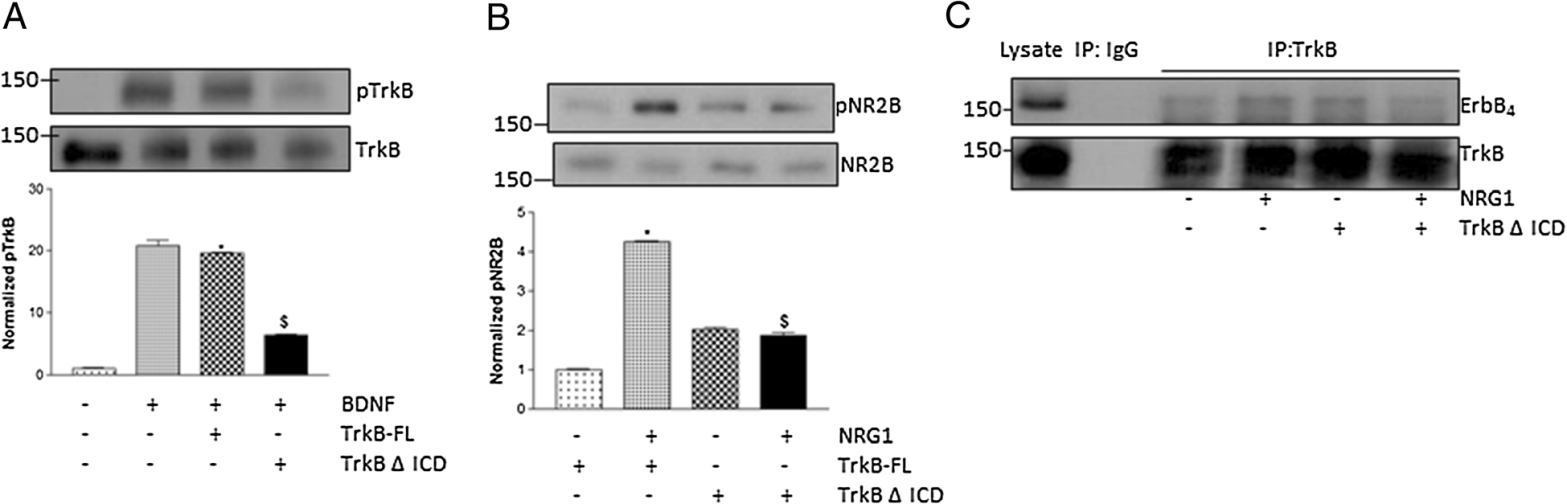
**Intracellular domain of TrkB was required for NRG1 induced NR2B activation. (A)** BDNF treatment increased TrkB activation in cortical neurons transfected with full-length TrkB (TrkB-FL), but not in neurons transfected with TrkB lacking intracellular domain (TrkB ∆ ICD). The upper panel shows a representative autoradiogram of pTrkB and TrkB, and the lower panel represents normalized pTrkB values. Data represent mean ± SEM. (n = 3). *P < 0.05 vs control; ^$^P < 0.05 vs BDNF treatment **(B)** NRG1 failed to activate NR2B in neurons transfected with TrkB ∆ ICD. The upper panel shows a representative autoradiogram of pNR2B and NR2B, and the lower panel represents normalized pNR2B values. Results are mean ± SEM. (n = 3) *P < 0.05 vs TrkB-FL; ^$^P < 0.05 vs NRG1 + TrkB-FL. **(C)** Immunoprecipitation (IP) with the anti-TrkB antibody or control IgG antibody followed by blotting with the anti-ErbB4 or anti-TrkB antibody was performed in NRG1-treated cortical neurons transfected with TrkB lacking intracellular domain (TrkB ∆ ICD).

The observed role of TrkB phosphorylation in NRG1-induced NR2B activation suggested an intracellular contribution to TrkB-ErbB4 interaction. Blockade of TrkB phosphorylation may inhibit receptor internalization as TrkB phosphorylation is needed for TrkB internalization [23]. To examine whether the intracellular domain of TrkB is involved in TrkB-ErbB4 interaction, mutant of TrkB lacking the intracellular domain (TrkB ∆ ICD) was used. The transfection efficiency was determined by quantification of the percentage of GFP-positive cells relative to all cells in the culture. We found 30-40% transfection efficiency in our experiments. The activity of TrkB ∆ ICD was confirmed in cultured cortical neurons. Treatment of neurons with BDNF stimulated TrkB activity in neurons expressing full length TrkB, but not in neurons transfected with TrkB ∆ ICD (Figure 5A). In addition, we did not find any significant increase in NR2B activation in neurons transfected with TrkB ∆ ICD (Figure 5B). To find out whether TrkB intracellular domain is necessary for NRG1-induced interaction between TrkB and ErbB4, we transfected neurons with TrkB ∆ ICD. NRG1-induced increase in TrkB-ErbB4 interaction was inhibited in neurons expressing TrkB ∆ ICD indicating the role of intracellular domain of TrkB in NRG1-induced TrkB-ErbB4 interaction (Figure 5C).

### Reduced TrkB/ErbB4 interaction in the prefrontal cortex of schizophrenia subjects

**Figure 6:**
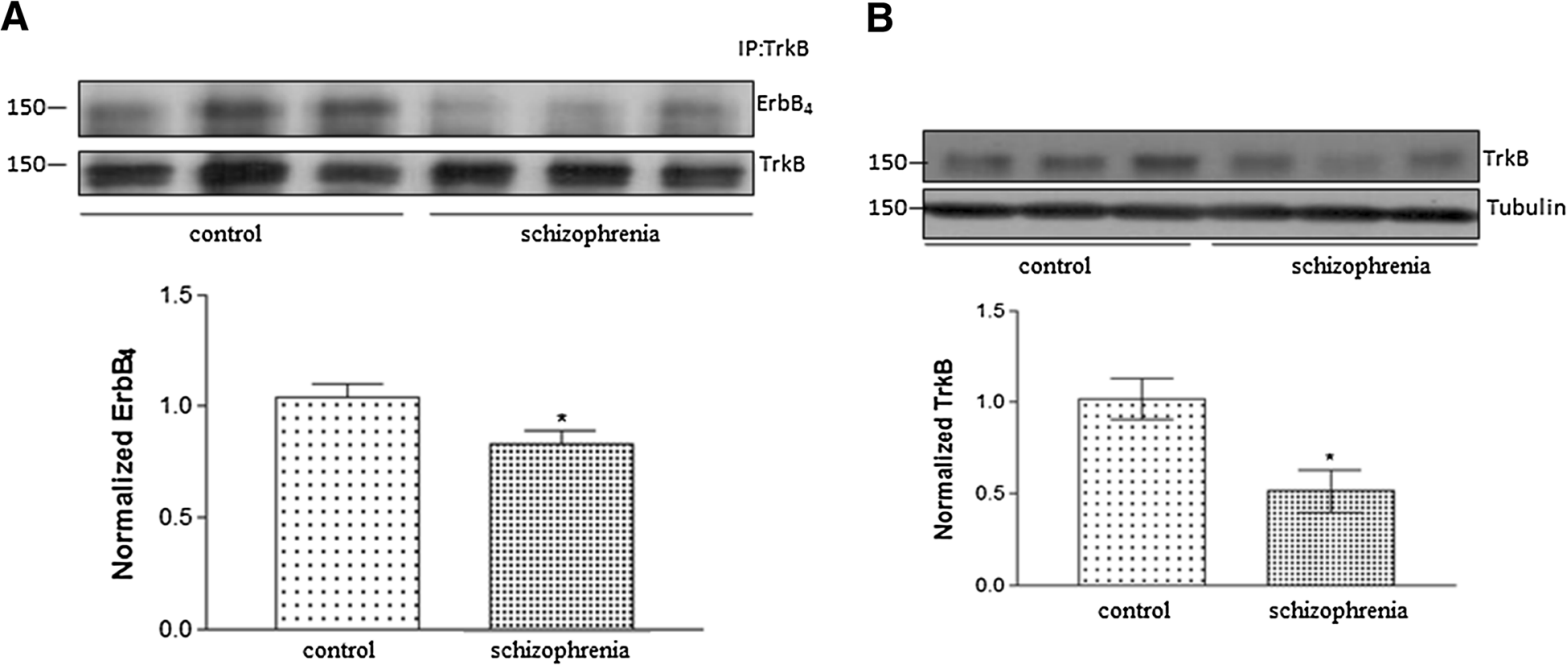
**Decrease in TrkB-ErbB4 interaction in the prefrontal cortex of schizophrenia subjects. (A)** Immunoprecipitation (IP) with the anti-TrkB antibody followed by blotting with the anti-ErbB4 or anti-TrkB was performed in prefrontal cortex samples from control (N = 15) and schizophrenia (N = 15) subjects. Representative immunoblots and quantification of normalized ErbB4 are shown. **(B)** Decrease in TrkB protein levels in the prefrontal cortex of schizophrenia subjects as determined by western blotting with anti-TrkB antibody. The upper panel shows a representative autoradiogram of TrkB and β-tubulin, and the lower panel represents normalized TrkB values. Data represent mean ± SEM. (N = 15 for schizophrenia and N = 15 for control subjects). *P < 0.05 vs controls.

Since both BDNF and NRG1 are implicated in the pathophysiology of schizophrenia, we examined whether the interaction between TrkB and ErbB4 is altered in schizophrenia. Following immunoprecipitation with TrkB, prefrontal cortical lysates from schizophrenia and control subjects were examined for ErbB4 levels. We found a decrease in TrkB/ErbB4 interaction in the prefrontal cortex of schizophrenia subjects as compared to controls (Figure 6A). In addition, we found a significant reduction in TrkB protein levels in the prefrontal cortex of schizophrenia subjects (Figure 6B).

## Discussion

We have shown that TrkB inhibition suppressed NRG1-stimulated NR2B phosphorylation via PLC signaling in cortical neurons before synaptogenesis. NRG1 treatment increased BDNF release from neurons and a BDNF-neutralizing antibody inhibited NRG1-induced NR2B activation. TrkB interacted with ErbB4 in neurons and the NRG1-induced increase in TrkB-ErbB4 interaction was decreased following TrkB inhibition.

Our data illustrate the interaction between two signaling pathways (BDNF and NRG1), which are well studied for their roles in synaptic plasticity and in the pathophysiology of many neuropsychiatric disorders including schizophrenia. Previously, independent studies have shown that both NRG1 and BDNF activate NR2B signaling in cortical neurons during or after synaptogenesis [7],[24]. We now show that TrkB is essential for NRG1 to activate NR2B in neurons before synaptogenesis. It has been shown previously that NRG1 activates ErbB4 and its interaction with PLCγ in neurons [25], and NR2B has been shown to bind to the SH domains of PLCγ [22]. Thus, PLCγ is probably the mediator of NRG1-induced NR2B phosphorylation in neurons. This conclusion is strongly supported by the present findings showing a robust reduction in phosphoNR2B levels when PLCγ activity was inhibited in NRG1-treated cells.

**Figure 7:**
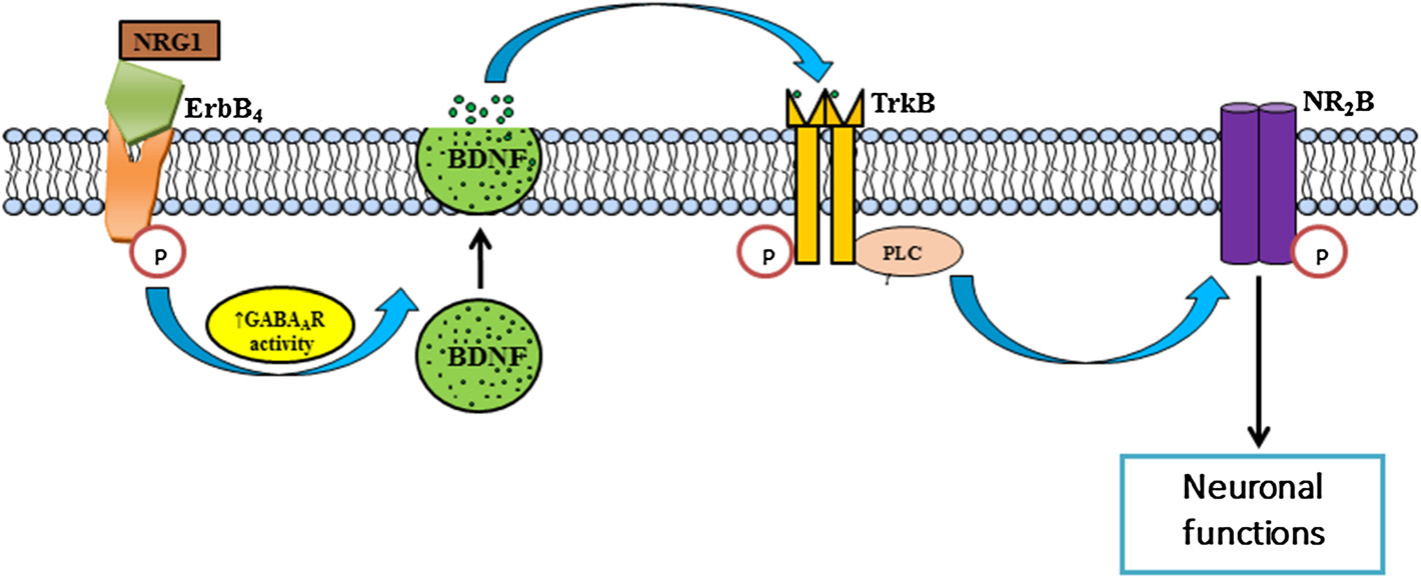
**BDNF/TrkB signaling regulates NR2B phosphorylation by NRG1 in immature cortical neurons.** NRG1-induced activation of its receptor ErbB4 on GABAergic interneurons results in an increased release of GABA. GABA_A_R activation, possibly via an autocrine manner in immature neurons leads to release of BDNF, and stimulation of signaling cascades, including TrkB and PLCγ. This leads to the activation of NR2B, providing the machinery required for various neuronal functions before synaptogenesis. Whether NRG1-induced GABA_A_R activation occurs in mature cortical neurons via autocrine and/or paracrine manner needs further investigation. In the brain, NRG1 [32] and BDNF [12] are primarily expressed in neurons and astrocytes *in vivo*.

NRG1 acutely activates NR2B phosphorylation via activation of ErbB4 in neurons [7]. The loss of TrkB function results in the reduction in NR2B activation following NRG1 exposure and that this is caused by alterations in BDNF levels. Thus, it is possible that the activation of ErbB4 by NRG1 contributes to NR2B phosphorylation by inducing the GABA_A_R-mediated release of BDNF from neurons, which then act on TrkB-expressing neurons promoting NR2B activation via PLCγ (Figure 7). It has been shown that ErbB4 signaling regulates BDNF expression, and the loss of ErbB4 signaling results in reduced BDNF expression in mice [26]. The lack of effect of BAPTA-AM on NRG1-induced TrkB phosphorylation suggests that NRG1 induces TrkB activation via calcium independent process. It has been previously shown that BDNF/TrkB signaling induces CREB phosphorylation in cortical neurons via MAP kinase pathway, but not through calcium-dependent process [27],[28]. Although inhibition of BDNF function suppresses NRG1-induced NR2B phosphorylation, the cellular localization of the above events is important. It has been shown that ErbB4 [29] and TrkB [30] are mostly expressed in parvalbumin (PV)-positive interneurons. Moreover, NMDARs expressed by the PV-positive fast spiking interneurons play a fundamental role in the maintenance of normal GABAergic function, and NMDAR hypofunction in PV neurons resulted in many behavioral and neurochemical abnormalities in mice homologous to schizophrenia [31].

We found a robust increase in TrkB-ErbB4 interaction following NRG1 treatment, which was suppressed by TrkB inhibition. It is possible that TrkB activation is necessary for the interaction of TrkB with ErbB4. Furthermore, the intracellular domain of TrkB is required for ErbB4 interaction. A significant reduction in TrkB-ErbB4 interaction found in the prefrontal cortex of schizophrenia subjects could be due to the decrease in TrkB expression previously reported in this brain region of schizophrenia subjects (17). We found that NRG1 treatment stimulates TrkB phosphorylation in neurons, which was inhibited by a BDNF neutralizing antibody, and BDNF inhibition abolished NRG1-induced increase in TrkB-ErbB4 interaction. NRG1-induced ErbB4 activation is known to increase GABA release [33]. Our findings indicate that GABA_A_R activation could be a possible mechanism for NRG1-induced increase in BDNF/TrkB signaling.

Both NRG1 and BDNF play important role in neurodevelopment and synaptic plasticity. Since NRG1 and BDNF are also present in non-neuronal cells such as oligodendrocytes and astrocytes, it is important to identify whether the processes similar to the ones found in the current study are functioning in those cell types. Moreover, it will be interesting to examine whether TrkB-ErbB4 interaction plays any role in GABAergic function in interneurons. The signaling mechanism described in this study, in which the regulation of NR2B activation by NRG1 is mediated by BDNF/TrkB signaling, may function in developing neural networks to enable NRG1 to modulate synaptogenesis, and growth of dendrites and axons prior to the formation of functional synapses. It will also be important to determine the extent to which this mechanism persists in the adult nervous system and contributes to the regulation of synaptic plasticity and cognitive function by NRG1 and BDNF. Given the alterations in NRG1 and BDNF signaling pathways result in neuronal dysfunctions as well as the implication of NMDARs in neuroplasticity, our findings on the role of TrkB in NRG1-stimulated NR2B phosphorylation could be of relevance to many neurodevelopmental disorders, as NRG1 and BDNF signaling pathways have been implicated in autism and schizophrenia.

## Materials and methods

### Animals

Timed pregnant CD-1 mice were purchased from Charles River Laboratories (Wilmington, MA, USA). TrkB knockout (C57BL/6; TrkB^−/−^) were provided by Dr. Barbara Rohrer, Medical University of South Carolina, Charleston, SC and the colony was maintained in our animal housing facility at the Georgia Regents University. All experiments were done in compliance with Georgia Regents University animal welfare guidelines.

### Time pregnancy and genotyping

TrkB^−/−^ mice were bred on C57BL/6 backgrounds and their offspring were genotyped at embryonic day 16 (E16) by PCR of tail biopsy DNA (DNeasy kits; Qiagen). PCR reaction was performed over 35 cycles using GoTaq® Green Master Mix (Promega). Primers utilized were as follows: *trkb-n2*: 5′-ATGTCGCCCTGGCTGAAGTG; *trkbc8*: 5′-ACTGACATCCGTAAGCCAGT; *pgk3-1*: 5′-GGTTCTAAGTACTGTGGTTTCC. Annealing temperature was set at 60°C. The products of the PCR reaction were visualized using agarose gel electrophoresis.

### Cell culture

Primary cortical neurons were prepared at E16. Neurons were cultured in Neurobasal medium containing, B27 supplement, 10% Fetal Bovine Serum (FBS), penicillin/streptomycin mixture of antibiotics and Glutamax for 4 days. The following pharmacological treatments were used: Neuregulinβ1 (Prospec, Israel) at 5 nM [34]; the Trk inhibitor, K252a (Tocris Biosciences, Minneapolis) at 100 nM; the ErbB4 inhibitor, AG1478 (Tocris) at 5 μM; PLC inhibitor, U73122 (Tocris) at 2 μM; BAPTA-AM (Tocris) at 50 μM; mouse anti-BDNF neutralizing antibody (1 μg/ml) [35]; GABA agonist, muscimol (Tocris) at 50 μM and GABA_A_ receptor antagonist, picrotoxin (Tocris) at 100 μM. Inhibitor, agonist or antagonist was added 20 or 30 min prior to NRG1 treatment.

### Immunoblotting

Cells were lysed in ice-cold radioimmununoprecipitation assay (RIPA) buffer (Tecnova) containing 150 mM NaCl, 1% Triton X-100, 1% sodium deoxycholate, 0.1% SDS, 50 mM Tris–HCl (pH 7.5), 2 mM EDTA (pH 8.0), 5 mM NaF, 2 mM Na3VO4, 1% protease inhibitor cocktail (Sigma), and 1 mM phenylmethylsulfonyl fluoride. The protein concentration was quantified using a BCA Protein Assay Kit (Sigma). Equal amounts of protein were resolved in SDS–polyacrylamide gels and transferred electrophoretically onto a nitrocellulose membrane (Bio-Rad). Blots were incubated with primary antibodies overnight at 4°C. After washing with 1× PBS and blocking with 5% milk in 1× PBS, blots were incubated with HRP-conjugated anti-rabbit or anti-mouse secondary antibody (Santa Cruz Biotechnology) for 1 h, followed by developing with the ECL Plus Western Blotting Detection System (GE Healthcare). Chemiluminescence signals were captured on autoradiographic blue films (Bioexpress). Films were scanned and the densitometric values for the proteins of interest were corrected using β-actin or β-tubulin with Image J Software (NIH). Primary antibodies were used at the following dilutions: anti-ErbB_4_ (1:1000, Cell Signaling), anti-pErbB_4_ (1:500, Cell Signaling), anti-TrkB (1:1000, Cell Signaling), anti-pTrkB (1:1000, kindly gifted by Dr Chao, New York University School of Medicine, New York) [36]-[38], anti-PLCγ (1:500, Santa Cruz Biotechnology Inc.), anti-pPLCγ (1:500, Cell Signaling), anti-PKC ζ (1:500, Santa Cruz Biotechnology Inc.), anti-pPKC ζ (1:500, Cell Signaling), anti-NR2B (1:500, Novus Biologicals), anti-pNR2B (1:500, Millipore), anti-BDNF (1:200, Santa Cruz Biotechnology Inc.), anti-βtubulin (1:5000, Cell Signaling) and anti-βactin (1:5000, Sigma). For immunoprecipitation, 300 μg of proteins were pre-cleared for 1 h with 30 μl of PureProteome Protein A and G Magnetic Beads (Millipore), followed by incubation overnight at 4°C in the presence of the primary antibody. The immunoprecipitated proteins were subjected to immunoblotting for the detection of the coprecipitated protein.

### Immunofluorescence staining

For immunofluorescence staining of primary cortical neurons, cultured neurons at DIV4 were permeabilized 5 min with 0.2% v/v triton-X100 and blocked for 1 hr at room temperature with 5% normal donkey serum. Anti-pErbB4 (1:1000) and anti-PV(1:2000; Swant) staining was performed overnight at 4°C followed by rinsing with PBS and incubation for 1 hr at room temperature with Cy2 and Cy3 conjugated secondary antibodies (Jackson Immunoresearch). Cells were then washed and mounted using ProLong® Gold Antifade Mountant containing DAPI (Molecular Probes). Images were taken using a LCM confocal microscope (Zeiss).

### Duolink proximity ligation assay (PLA)

The PLA was performed using Duolink *In Situ* reagents (Sigma). The cortical neurons (~2 × 10^4^ cells/well) were seeded into 24-well plate on poly-D-lysine coated cover slip. After treatment, the cortical neurons were washed twice with ice-cold 1 × PBS and fixed with 4% paraformaldehyde and 120 mM sucrose in PBS at room temperature for 20 min. After permeabilization the cells were incubated in the blocking buffer (provided with the kit) overnight at 37°C in a humidified chamber. The cells were incubated with the primary antibodies, anti-TrkB (1:500, Cell Signaling) and anti-ErbB4 (1:500, Cell Signaling) diluted in the antibody diluents for 2 hours at room temperature followed by washing in Buffer A (supplied with the kit) 3 times for 15 minutes and incubation with the PLA probes for one hour at 37°C in a humid chamber. The antibodies were omitted in the PLA control group. The cells were again subjected to a 10 minute wash and a 5 minute wash in Buffer A. The ligation reaction was carried out at 37°C for one hour in a humid chamber followed by a 10 and 5 minute wash in Buffer A. The cells were then incubated with the amplification-polymerase solution for two hours at 37°C in a darkened humidified chamber. After washing with 1x Buffer B (supplied with the kit) for 10 minutes followed by a 1 minute wash with 0.01X buffer B the cells were mounted using the mounting media supplied with the kit. Images were collected using Zen 2012 lite imaging software from several fields of view per experiment. The number of PLA signals per cell (indentified as red spots) was counted from three Z-plane images using ImageJ (NIH).

### Brain-derived neurotrophic factor immunoassay

Brain-derived neurotrophic factor was measured with a conventional sandwich ELISA using the BDNF Emax Immuno-Assay System (Promega, Madison, WI, USA) according to the protocol of the manufacturer.

### TrkB deletion plasmids

pBiFC-TrkB-FL and pBiFC-TrkB-∆-ICD constructs were kindly provided by Dr Maruyama, Okinawa Institute of Science and Technology, Japan [39]. These constructs were transfected into primary cortical neurons using Effectene Transfection Reagent (Qiagen) 48 h before NRG1 treatment.

### Small interfering RNA (siRNA)

We used 19 nt siRNA (GCACAUAAAUUUCACACGA, M-048017-01-0005) named Ntrk2 for mouse TrkB and 19 nt siRNA (GCAAGAAGUUCCUCCAGUA) for mouse PLCγ (M-040978-01-0005) from Dharmacon Research Inc. The mouse control siRNA used was 19 nt quadruplex with two 3′ overhanging nucleotides (D-001206-13, Dharmacon Research Inc.). Transfection of both siRNAs (50 nM) was performed in cultured cortical neurons using Amaxa 4D-Nucleofector Protocol (Lonza) 48 h before the NRG1 treatment.

### Postmortem samples

We obtained postmortem prefrontal cortex samples from schizophrenia (N = 15) and control (N = 15) subjects from the Human Brain and Spinal Fluid Resource Center (Los Angeles, California, United States). Description on the demographic details of samples is published elsewhere [40]. The samples were shipped frozen and stored at −80°C until analysis. Grey matter was removed from a 1.5–2.0 cm thick coronal slab of the frontal cortex anterior to the corpus callosum and the prefrontal cortex was dissected [41]. Tissue was homogenized in a homogenizing buffer containing 20 mM Tris–HCl (pH 7.4), 2 mM EGTA, 5 mM EDTA, 1.5 mM pepstatin, 2 mM leupeptin, 0.5 mM phenylmethylsulfonyl fluoride, 0.2 U/ml aprotinin, and 2 mM dithiothreitol. The homogenate was centrifuged at 15,000 rpm for 15 min at 4°C. Protein concentration in the supernatant was determined with BCA Reagent.

### Statistical analysis

Quantified data are presented as mean ± SEM and analyzed by GraphPad PRISM. Statistical comparisons between two groups were made using t tests. Comparisons among multiple groups were made using one-way or two-way ANOVA, with Bonferroni’s post hoc analyses to identify significant differences between groups. The probability (*p*) values of less than 5% were considered significant.

## Competing interest

The authors declare that they have no competing interests.

## Authors’ contributions

AP designed the study. CDP performed the experiments and analyzed the data. AP wrote the manuscript. Both authors approved the final version of the manuscript.
